# Carpal Tunnel Syndrome at the Intersection of Internal Medicine, Gastroenterology, and Neurology: A Thorough Examination

**DOI:** 10.3390/jcm14197022

**Published:** 2025-10-03

**Authors:** Sefer Aslan, Hüsniye Aylin Dikbaş, Ali Muhtaroğlu, Ersin Kuloğlu, Gökhan Aydın, Ahmet Cumhur Dülger

**Affiliations:** 1 Department of Internal Medicine, Faculty of Medicine, Giresun University, Giresun 28200, Turkey; ersinkuloglu.28@hotmail.com; 2 Department of Neurology, Faculty of Medicine, Giresun University, Giresun 28200, Turkey; husniyeaylin@yahoo.com; 3 Department of General Surgery, Faculty of Medicine, Giresun University, Giresun 28200, Turkey; alimuhtarogluu@gmail.com; 4 Department of Gastroenterology, Faculty of Medicine, Giresun University, Giresun 28200, Turkey; gokhanaydina@hotmail.com (G.A.); acdulger@gmail.com (A.C.D.)

**Keywords:** carpal tunnel syndrome, electromyography, gastrointestinal endoscopy, systemic inflammation

## Abstract

**Background/Objectives:** This study was designed to investigate the potential clinical, biochemical, haematological, and pathological associations of carpal tunnel syndrome through a multidisciplinary approach encompassing the fields of internal medicine, gastroenterology, and neurology. **Methods:** The study group (CTS-positive) comprised 265 patients who presented with dyspeptic complaints and underwent upper gastrointestinal endoscopy, gastric antrum biopsy, electromyography, and comprehensive biochemical and haematological analyses. A control group of 265 patients with similar symptoms but without CTS was selected for comparison. A comparative analysis was conducted on clinical findings, gastric biopsy results, and biochemical and haematological parameters. **Results:** There were no significant differences in age, gender distribution, or gastric biopsy findings (*Helicobacter pylori*, intestinal metaplasia, atrophy, and dysplasia) between the CTS-positive and CTS-negative groups. However, significant biochemical differences were identified, including elevated calcium and reduced magnesium levels in CTS-positive patients. Haematological evaluations revealed higher lymphocyte, eosinophil, basophil, erythrocyte, haemoglobin, and haematocrit levels, along with reduced neutrophil-to-lymphocyte ratios and red blood cell distribution widths in the CTS-positive group. Further analysis in the form of correlation and logistic regression analyses provided further confirmation of the association of elevated calcium, haemoglobin, and lymphocyte levels with increased risk of CTS. **Conclusions:** This multidisciplinary study identifies significant associations between CTS and specific biochemical and haematological parameters, notably calcium-magnesium imbalance and erythrocyte indices. These findings suggest underlying biological interactions that may guide future diagnostic and therapeutic strategies for patients with carpal tunnel syndrome.

## 1. Introduction

Carpal Tunnel Syndrome (CTS) is a prevalent peripheral nerve entrapment disorder, characterised by the compression of the median nerve at the wrist as it passes through the carpal tunnel. From a clinical perspective, the condition known as CTS presents with a range of symptoms including numbness, tingling, pain, and functional impairment. These symptoms predominantly affect the thumb, index finger, middle finger, and the radial half of the ring finger. CTS has been demonstrated to exert a considerable effect on patients’ quality of life, with the capacity to impair both daily activities and work productivity [1].

The underlying causes of CTS are multifactorial in nature and include repetitive hand motions, wrist trauma, rheumatoid arthritis, diabetes mellitus, hypothyroidism, obesity, and hormonal changes such as those occurring during pregnancy or menopause. Recent studies have also suggested potential systemic factors, including biochemical and haematological imbalances, that may influence its pathogenesis [2].

CTS is known to manifest in a unilateral or bilateral pattern, each of which is characterised by distinct clinical features. Bilateral involvement is a common occurrence, manifesting with variable severity levels on each side. Although bilateral CTS is commonly associated with systemic factors such as endocrine disorders and inflammatory conditions, unilateral CTS often results from local anatomical or occupational factors. Distinguishing between right, left, or bilateral involvement may thus have diagnostic and therapeutic implications [3].

Dyspepsia, a term given to a range of symptoms including epigastric pain, bloating, early satiety and nausea, often leads to upper gastrointestinal endoscopy as part of the diagnostic process. Patients presenting with concurrent dyspeptic symptoms and CTS pose a clinical challenge due to the potential for overlapping systemic factors. The management of these patients necessitates an integrative approach that addresses both neurological and gastrointestinal symptoms [4].

The conventional treatment for CTS comprises conservative interventions such as wrist splinting, anti-inflammatory medications, physical therapy, and corticosteroid injections. Surgical decompression is indicated for cases that are severe or resistant to other forms of treatment [5]. The management of dyspeptic symptoms is chiefly achieved through lifestyle modifications, the administration of proton pump inhibitors (PPIs), the utilisation of histamine H2-receptor antagonists, the employment of prokinetic agents, and, when indicated by biopsy findings, Helicobacter pylori (*H. pylori*) eradication therapy [6].

Despite their apparent distinctiveness, treatments for dyspepsia and CTS have similarities at the systemic level, particularly in patients with metabolic, inflammatory, or nutritional imbalances. For instance, the management of systemic inflammation, electrolyte imbalances (particularly calcium and magnesium), and nutritional deficiencies may be advantageous in addressing both conditions [7]. The exploration of these overlapping factors has the potential to refine therapeutic outcomes and to provide insights into shared underlying pathophysiological mechanisms.

The objective of this study is to explore the potential clinical, biochemical, haematological, and pathological associations of CTS in patients presenting with dyspeptic complaints. A more profound understanding of these associations has the potential to facilitate the development of more precise diagnostic approaches, optimised therapeutic strategies, and ultimately, enhanced patient care through interdisciplinary collaboration across internal medicine, gastroenterology, and neurology.

## 2. Methods

The study protocol was approved by the local ethics committee of our hospital on 22 January 2025, with the decision numbered 22 January 2025/08. The study was retrospective, so we did not require informed consent. This study was conducted in accordance with the relevant ethical principles of the Declaration of Helsinki/Finland, revised in 2013.

This study included a total of 530 patients who presented to internal medicine, general surgery, and gastroenterology outpatient clinics between January 2022 and December 2024 with dyspeptic complaints. All patients underwent detailed clinical evaluations and upper gastrointestinal endoscopy. Gastric antrum biopsies were systematically collected from all patients during endoscopic procedures, and histopathological assessments were performed to evaluate the presence of intestinal metaplasia, atrophy, and dysplasia. The presence of *H. pylori* was evaluated pathologically through the use of hematoxylin and eosin staining. Endoscopy was performed by gastroenterologists, with the mucosal changes evaluated using high-resolution electronic endoscopy equipment (Fujifilm^®^, Tokyo, Japan, EPX3500 HD series).

Patients were stratified into two distinct groups based on electromyography (EMG) findings: those diagnosed with carpal tunnel syndrome (CTS-positive group, *n* = 265) and a control group without CTS (CTS-negative group, *n* = 265). The control group was matched for dyspeptic symptom presentation but lacked Electromyography (EMG) evidence of median nerve entrapment.

All participants underwent comprehensive biochemical evaluations, including measurements of glucose, urea, creatinine, alanine aminotransferase (ALT), aspartate aminotransferase (AST), electrolytes (sodium, potassium, chloride), lipid profiles (cholesterol, triglycerides, HDL, LDL), thyroid-stimulating hormone (TSH), vitamins (folate, vitamin B12), protein, albumin, calcium, magnesium, and uric acid levels. Blood samples were collected following standard venipuncture protocols and analysed using automated laboratory methods.

Haematological analyses comprised complete blood counts (CBC), measuring leukocytes, neutrophils, lymphocytes, monocytes, eosinophils, basophils, erythrocytes, haemoglobin, haematocrit, mean corpuscular volume (MCV), red blood cell distribution width (RDW), platelets, mean platelet volume (MPV), and platelet distribution width (PDW). Additionally, systemic inflammatory indices were calculated, including the neutrophil-to-lymphocyte ratio (NLR), platelet-neutrophil/lymphocyte index, platelet-to-lymphocyte ratio (PLR), systemic immune inflammation index (SII), C-reactive protein-to-albumin ratio (CAR), and albumin-to-globulin ratio (AGR).

CTS severity was classified using EMG criteria into mild, moderate, or severe categories, and involvement was categorised as unilateral (right or left) or bilateral. Patients’ demographic characteristics, including age and gender, were recorded for comparative analysis.

### Statistical Analysis

Data analysis was performed using the Statistical Package for the Social Sciences (SPSS) 26.0 Statistics software package. The suitability of numerical variables for normal distribution in CTS-positive and CTS-negative patients who underwent EMG and endoscopy was determined by examining skewness and kurtosis values. Data from patients with a normal distribution are denoted by the letter t/F, while data from patients with a non-normal distribution are denoted by the letter z/H. The reference value for normal distribution is ±1.96. The Chi-square test was used to compare the descriptive characteristics and gastric biopsy findings of CTS-positive and CTS-negative individuals. The Independent Sample *t* Test or Mann–Whitney U test was used to compare the age and laboratory values of CTS-positive and CTS-negative individuals. The Spearman Correlation test was used to examine the relationship between CTS positivity and clinical, biopsy, biochemical, haematological, and inflammatory findings. The correlation coefficient was evaluated as low (0.00–0.30), moderate (0.30–0.70), and high (0.70–1.00) levels of association. Variables that could influence CTS positivity were evaluated using Single Logistic Regression analysis. ROC analysis was performed to predict the likelihood of CTS. Significance levels of 0.05 and 0.01 were considered in all analyses. In this study, the size of the area under the ROC curve is statistically significant in predicting the probability of patients having or not having CTS. If ROC analyses show no ability to distinguish between patients with and without CTS, the expected value of the area under the ROC curve is 0.50. In a perfect test, this value is expected to be 1.00. The values under the curve can be interpreted as follows: 0.90–1.00 = excellent, 0.80–0.90 = good, 0.70–0.80 = moderate, 0.60–0.70 = poor, and 0.50–0.60 = inadequate [8]. In this context, it was observed that all variables had a significant effect on predicting the probability of patients having or not having KTS (*p* < 0.05).

## 3. Results

The demographic comparison between carpal tunnel syndrome (CTS)-positive and CTS-negative groups revealed no significant differences in age or gender distribution, with both groups exhibiting a similar female predominance (*p* > 0.05). Gastric biopsy results indicated comparable rates of *H. pylori* infection, intestinal metaplasia, atrophy, and dysplasia across both CTS-positive and CTS-negative cohorts, with no statistically significant differences observed (*p* > 0.05). Table 1 presents a comparison of the descriptive characteristics of patients undergoing endoscopy with CTS-positive (*n* = 265) and negative (*n* = 265) individuals, along with gastric biopsy findings.

Biochemical parameter analyses demonstrated significant differences in calcium and magnesium levels. Specifically, calcium concentrations were significantly elevated in the CTS-positive group compared to the CTS-negative group (*p* < 0.05), whereas magnesium levels were significantly lower in CTS-positive individuals (*p* < 0.05). No significant differences were observed for other biochemical parameters, including electrolytes (sodium, potassium) albumin, uric acid and CRP (*p* > 0.05). Table 2 compares the biochemical parameter levels of CTS-positive and CTS-negative patients who underwent endoscopy.

Haematological assessments revealed notable distinctions. Lymphocyte, eosinophil, basophil, erythrocyte, haemoglobin, and haematocrit levels were significantly higher in the CTS-positive group (*p* < 0.05). Conversely, the CTS-positive patients exhibited significantly lower neutrophil-to-lymphocyte ratios (NLR) and red blood cell distribution widths (RDW) (*p* < 0.05). No significant differences were found in leukocyte count, monocyte count, mean corpuscular volume (MCV), platelet count, mean platelet volume (MPV), and platelet distribution width (PDW) (*p* > 0.05). Table 3 compares the haematological findings and inflammatory indices of CTS-positive and CTS-negative patients who underwent endoscopy.

Correlation analysis showed significant, albeit low-level, positive correlations between CTS positivity and calcium (r = 0.165), haemoglobin (r = 0.165), haematocrit (r = 0.160), erythrocyte (r = 0.132), lymphocyte (r = 0.107), basophil (r = 0.113), and eosinophil (r = 0.087) levels (*p* < 0.05). Negative correlations were observed with magnesium levels (r = −0.127), NLR (r = −0.147), and RDW (r = −0.134) (*p* < 0.05). No correlations were identified between CTS positivity and age, gender, gastric biopsy findings, and most other biochemical parameters. In Table 4, the relationships between CTS positivity and diagnostic, gastric biopsy, biochemical, haematological, and inflammatory parameters in patients who underwent endoscopy were evaluated using correlation analysis.

Analysis according to CTS direction (right, left, bilateral) showed no significant differences among groups regarding gender distribution, CTS severity, or gastric biopsy findings (Helicobacter pylori, intestinal metaplasia, atrophy, dysplasia) (*p* > 0.05). However, biochemical parameters revealed significantly elevated LDL cholesterol and albumin levels in the right CTS group and higher chloride levels in the left CTS group (*p* < 0.05). Uric acid levels were notably higher in the right CTS group, although not significantly (*p* > 0.05). Haematological analyses indicated higher neutrophil counts and mean corpuscular volumes in the right CTS group and higher erythrocyte distribution widths in the left CTS group, all statistically significant (*p* < 0.05). Differences in inflammatory indices among CTS directions approached significance, notably the C-reactive protein-to-albumin ratio (CAR, *p* = 0.053). Table 5 presents a comparison of clinical, biochemical, haematological, and inflammatory findings among CTS-positive patients according to CTS direction (right, left, and bilateral).

Comparison based on CTS severity (mild, moderate, severe) revealed no statistically significant differences among the groups regarding age, gender distribution, or gastric biopsy findings (Helicobacter pylori, intestinal metaplasia, atrophy, dysplasia) (*p* > 0.05). Similarly, biochemical and inflammatory parameters did not significantly differ among groups stratified by severity (*p* > 0.05). Haematological evaluations showed no significant variations across mild, moderate, or severe CTS groups (*p* > 0.05). Table 6 presents a comparison of CTS-positive patients according to CTS severity (mild, moderate, severe) in terms of clinical, gastric biopsy, and age variables.

Logistic regression analyses indicated a significant association between CTS positivity and calcium (OR = 2.00), haemoglobin (OR = 1.24), haematocrit (OR = 1.08), erythrocyte count (OR = 1.67), and lymphocyte levels (OR = 1.32) (*p* < 0.05). Negative associations were identified with NLR (OR = 0.83) and RDW (OR = 0.85), indicating protective effects against CTS positivity (*p* < 0.05). In Table 6, variables that may affect CTS positivity were evaluated using single logistic regression analysis. The predictive power of each independent variable for CTS positivity is expressed by the odds ratio (Exp(B)) and *p*-value. Additionally, the explanatory power of each model is indicated by the R^2^ (pseudo R^2^) value.

Receiver Operating Characteristic (ROC) analysis indicated that calcium, haemoglobin, haematocrit, lymphocyte levels, and erythrocyte counts had statistically significant predictive value for CTS positivity. However, inflammatory indices, including the platelet–neutrophil/lymphocyte index and NLR, demonstrated limited discriminatory power despite statistical significance (AUC values <0.60, *p* < 0.05).

**Figure 1:**
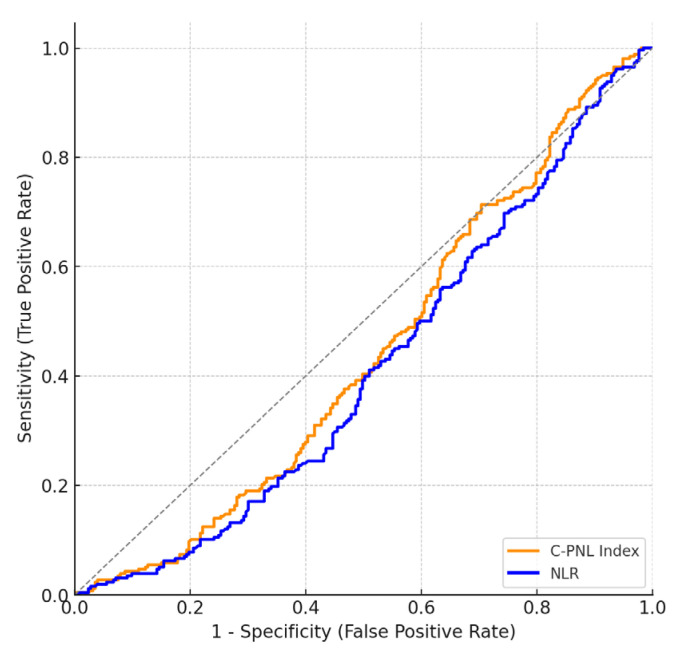
Results of the ROC Analysis Performed to Predict the Probability of Being a CTS Patient.

The results of the ROC analysis performed to estimate the probability of KTS are shown in Table 7 and Figure 1.

In this study, the predictive power of the Composite PLT–NEUT–Lymph Index and NLR variables in predicting KTS development was found to be statistically insufficient.

The area under the curve (AUC) for the Composite PLT–Nöt–Lymph Index was found to be 0.56, and this value was statistically significant (*p* = 0.025). The cutoff value determined for this index was 425.80, with sensitivity calculated as 60% and specificity as 50%. Similarly, the AUC value for the Neutrophil/Lymphocyte Ratio (NLR) was 0.59, with a significance level of *p* = 0.001. The cutoff value determined for NLR was 1.69, with sensitivity of 62% and specificity of 50%. Although both test variables were found to be statistically significant, the AUC values remaining below 0.60 indicate that these variables have limited discriminatory power in predicting the likelihood of being a CTS patient.

## 4. Discussion

This study provides a comprehensive examination of carpal tunnel syndrome (CTS) through a multidisciplinary process, integrating perspectives from the fields of internal medicine, gastroenterology, and neurology. The results demonstrate a significant association between CTS and specific biochemical and haematological parameters, such as elevated calcium and reduced magnesium levels, alongside altered erythrocyte indices. These findings align with and expand upon previous literature while introducing novel insights into the systemic dimensions of CTS.

In our study, the association between calcium levels and CTS, identified as a strong predictor in logistic regression analysis (OR = 2.00), is consistent with current hypotheses regarding the role of calcium homeostasis in nerve conduction and neuromuscular excitability. Previous studies have demonstrated that calcium balance is crucial for the functionality of peripheral nerves. It has been hypothesised that hypercalcemia may exacerbate nerve compression syndromes through mechanisms such as alterations in membrane potential and increased nerve excitability [9,10]. The present findings in patients diagnosed with CTS further support this association and highlight the clinical relevance of monitoring calcium levels, particularly in cases where systemic disorders coexist.

On the other hand, the observation of lower magnesium levels in CTS-positive patients is consistent with studies demonstrating the neuroprotective effects of magnesium, which regulates calcium flow and stabilises nerve membranes [11,12]. The dynamic relationship between magnesium and calcium, both of which are essential electrolytes, suggests that imbalances may play a role in CTS pathogenesis through mechanisms potentially involving nerve ischemia, inflammation, or oxidative stress [13].

The haematological findings obtained in this study, particularly the observation of increased levels of lymphocytes, eosinophils, basophils, erythrocytes, haemoglobin, and hematocrit in CTS patients, suggest that systemic inflammatory and hematopoietic mechanisms may contribute to the development of CTS [14]. Elevated lymphocyte levels may indicate a mild proinflammatory response, while increases in eosinophils and basophils may point to low-level allergic or immunological processes. Elevated erythrocyte parameters may reflect compensatory adaptations to increase oxygen transport due to chronic nerve compression. This observation is supported by previous studies reporting a link between erythrocytosis and chronic hypoxia in compressive neuropathies [15,16].

In a study conducted by Güneş et al., there was a positive correlation between CTS severity and NLR, age, and body mass index. A 1-unit increase in NLR was associated with an approximately 1.7-fold increase in the incidence of CTS [17]

The negative correlation identified between CTS and inflammatory markers such as the neutrophil-to-lymphocyte ratio (NLR) and red blood cell distribution width (RDW) provides a noteworthy contrast to prior reports linking elevated NLR with systemic inflammation and adverse outcomes in diverse diseases. These results imply that CTS, despite being a localised neuropathic condition, may not consistently reflect systemic inflammatory responses [18,19]. Therefore, further research is warranted to clarify the interplay between localised and systemic inflammation in the development of CTS.

This research is one of the earliest to explore the association between CTS and gastric abnormalities in individuals presenting with dyspeptic complaints. The lack of significant differences in gastric biopsy outcomes (such as *H. pylori* infection, intestinal metaplasia, atrophy, and dysplasia) suggests that although CTS and dyspepsia may coexist, their underlying pathophysiological mechanisms are likely distinct [20]. Nonetheless, the presence of shared risk factors—including metabolic disturbances and persistent low-grade inflammation—highlights the importance of adopting a comprehensive, multidisciplinary approach in the management of these patients [21,22].

Subgroup analyses based on CTS direction (right, left, bilateral) and severity (mild, moderate, severe) provide further granularity. The right-sided predominance of higher LDL cholesterol, albumin, and neutrophil levels, along with elevated erythrocyte distribution width in the left CTS group, suggests potential lateralised vascular or metabolic influences on CTS manifestation. However, the absence of significant differences in gastric findings or most systemic parameters across CTS severity categories indicates that while certain laboratory abnormalities are associated with CTS presence, they may not directly correlate with disease progression [23].

Despite the absence of a direct correlation between CTS and gastric pathology, the findings illustrate the significance of a systemic perspective in CTS evaluation and management. These results contribute valuable insights to the extant literature and underscore the need for prospective studies to explore the causal relationships and therapeutic implications of these associations.

### Study Limitations

The current study is subject to several limitations. The retrospective design of the study limits causal inference, and the lack of longitudinal data limits understanding of biochemical and haematological changes over time. Parameters such as PTH, fibrinogen/albumin ratio, and ionised calcium were studied in a limited number of cases in our study and were not statistically evaluated. The possibility of selection bias due to the inclusion of patients presenting only with dyspeptic complaints is a potential limitation of the study. Potential confounding factors such as occupational handedness, lifestyle factors, and genetic predisposition were not evaluated in the current study. Other factors to consider include the limitations of single-point measurements for laboratory parameters and the sole reliance on EMG for CTS classification. Furthermore, although associations between CTS, pan-immune inflammatory markers, and gastric pathology have been investigated, mechanistic associations have not been explored.

## 5. Conclusions

This multidisciplinary study demonstrates significant associations between carpal tunnel syndrome (CTS) and specific biochemical and haematological parameters, including elevated calcium levels, reduced magnesium levels, and altered erythrocyte indices. The study also notes the absence of a clear relationship between CTS and gastric pathology in patients with dyspeptic symptoms. The findings suggest that CTS may have systemic origins influenced by metabolic, haematological, and inflammatory factors, emphasising the necessity of a comprehensive, interdisciplinary approach to patient evaluation. Despite certain limitations, such as the retrospective design and lack of longitudinal follow-up, this study provides novel insights into the complex interplay between CTS and systemic health. These results contribute to the growing body of evidence supporting a broader perspective in the diagnosis and management of CTS and lay the basis for future prospective research to explore potential therapeutic targets and mechanisms.

## Figures and Tables

**Table 1:** Comparison of Descriptive Characteristics and Gastric Biopsy Findings of Carpal Tunnel Syndrome (CTS) Positive and Negative Individuals in Patients Who Underwent Electromyography (EMG) and Endoscopy.

Variables		CTS Negative (*n*:265)		CTS Positivity (*n*:265)		*p*
		Count	%	Count	%	
Gender	Female	201	75.8	202	76.2	1.000
	Male	64	24.2	63	23.8	
CTS Direction	Right	0	0.0	45	17.0	-
	Left	0	0.0	49	18.5	
	Bilateral	0	0.0	171	64.5	
CTS Severity	Mild	0	0.0	108	408	-
	Moderate	0	0.0	128	48.3	
	Severe	0	0.0	29	10.9	
*H. pylori*	Negative	161	60.8	164	61.9	0.858
	Positive	104	39.2	101	38.1	
Int. Metaplasia	Negative	235	88.7	234	88.3	1.000
	Positive	30	11.3	31	11.7	
Atrophy	Negative	238	89.8	248	93.6	0.157
	Positive	27	10.2	17	6.4	
Dysplasia	Negative	261	98.5	263	99.2	0.681
	Positive	4	1.5	2	0.8	
		Mean ± S.S. Med. (Min.–Max.)		Mean ± S.S. Med. (Min.–Max.)		
Age ^t^		59.25 ± 11.66 60 (28–90)		60.06 ± 10.86 60 (6–87)		0.408

Note: χ^2^: Chi-square test (categorical data); ^t^: Independent Sample *t*-test.

**Table 2:** Comparison of Biochemical Findings of CTS Positive and Negative Individuals in Patients Who Underwent EMG and Endoscopy.

Biochemical Findings	With CTS Negative (*n*:265)	With CTS Positivity (*n*:265)	*p*
	Ort. ± S.S Med. (Min.–Max.)	Ort. ± S.S Med. (Min.–Max.)	
Sodium ^z^	140.5 ± 4.4 141 (128–180)	140.77 ± 2.9 141 (128–150)	0.054
Potassium ^t^	4.41 ± 0.48 4.4 (2.8–6.2)	4.48 ± 0.43 4.4 (3.4–5.9)	0.073
Calcium ^z^	9.39 ± 0.68 9.5 (4.3–11)	9.6 ± 0.47 9.6 (7.5–11.4)	0.000 **
Magnesium ^z^	2.06 ± 0.57 2.01 (1.41–8.6)	1.97 ± 0.21 1.99 (1.36–2.71)	0.025 **
Albumin ^z^	43.59 ± 5.24 44.7 (16.2–52)	44.92 ± 3.06 45 (32–55)	0.102
Uric Acid ^t^	4.78 ± 1.37 4.6 (1.52–9.78)	4.76 ± 1.3 4.61 (1.96–9.93)	0.859
Phosphorus ^t^	3.61 ± 0.59 3.6 (2.18–5.3)	3.72 ± 0.56 3.7 (2.4–5)	0.146
CRP ^z^	12.69 ± 39.62 2.4 (0.06–357.46)	10.02 ± 42.58 2.16 (0.06–389.65)	0.151

Note: ** *p* < 0.05; ^z^: Mann–Whitney U test; ^t^: Independent Sample *t*-test.

**Table 3:** Comparison of Haematological Findings and Inflammatory Indexes of CTS Positive and Negative Individuals in Patients Who Underwent EMG and Endoscopy.

Biochemical Findings	With CTS Negative (*n*:265)	With CTS Positivity (*n*:265)	*p*
	Ort. ± S.S Med. (Min.-Max.)	Ort. ± S.S Med. (Min.-Max.)	
Lymphocyte ^z^	2.09 ± 0.77 2.01 (0.24–5.18)	2.24 ± 0.72 2.11 (0.76–6.09)	0.016 *
Eos ^z^	0.18 ± 0.15 0.14 (0–0.95)	0.2 ± 0.16 0.16 (0–1.11)	0.048 *
Head ^z^	0.04 ± 0.02 0.03 (0.01–0.11)	0.04 ± 0.02 0.04 (0.01–0.17)	0.013 *
RBC ^z^	4.44 ± 0.63 4.5 (1.77–6.71)	4.6 ± 0.48 4.63 (3.11–5.96)	0.003 **
HGB ^z^	12.4 ± 1.96 12.6 (3.72–16.8)	13.05 ± 1.5 13 (8.3–17.2)	0.000 **
HCT ^z^	38.11 ± 5.46 38.6 (12.2–52.3)	39.86 ± 4.03 39.8 (26.5–51.8)	0.000 **
RDW-CV ^z^	14.79 ± 5.62 14 (11.6–98.1)	13.9 ± 1.67 13.6 (11.3–22)	0.002 **
C-PNL Index ^z^	627.06 ± 507.71 479.52 (22.18–4141.96)	524.26 ± 460.56 428.55 (111–6189.07)	0.025 *
NL ^z^	2.62 ± 2.5 1.93 (0.3–19.63)	2.01 ± 1.52 1.7 (0.68–18.93)	0.001 **
PLR ^z^	137.51 ± 71.7 124.18 (5.7–654.17)	126.01 ± 49.7 116.76 (36.94–398.78)	0.122
SII ^z^	339.29 ± 357.01 232.31 (4.44–3316.85)	268.9 ± 317.58 200.87 (0–4332.35)	0.064
CAR ^z^	0.43 ± 1.87 0.05 (0–17.27)	0.17 ± 0.8 0.05 (0–10.92)	0.190
AGR ^z^	1.72 ± 0.35 1.74 (0.23–2.92)	1.78 ± 0.36 1.76 (0.94–4.23)	0.244

Note: * *p* < 0.05; ** *p* < 0.01. ^z^: Mann–Whitney U test. C-PNL Index: Composite Plate-let–Neutrophil-to-Lymphocyte Index.

**Table 4:** Evaluation of the Relationship Between CTS Positivity and Clinical. Biopsy. Biochemical. Haematological and Inflammatory Findings.

Variable	Number of Floors	CTS Positivity	Variable	Number of Floors	CTS Positivity	Variable	Number of Floors	CTS Positivity
Age	r	0.030	GGT	r	0.011	Mono#	r	−0.033
	p	0.488		p	0.825		p	0.456
Gender	r	−0.004	Cholesterol	r	−0.002	Eos#	r	0.087 *
	p	0.919		p	0.966		p	0.048
*H. pylori*	r	−0.012	Triglyceride	r	0.007	Bas#	r	0.113 *
	p	0.790		p	0.894		p	0.013
Int. Metaplasia	r	0.010	HDLK	r	0.034	RBC	r	0.132 **
	p	0.89		p	0.527		p	0.003
Atrophy	r	−0.07	LDLK	r	−0.029	HGB	r	0.165 **
	p	0.116		p	0.637		p	0.000
Dysplasia	r	−0.036	TSH	r	0.031	HCT	r	0.160 **
	p	0.413		p	0.513		p	0.000
Glucose	r	0.004	Ferritin	r	0.019	MCV	r	0.028
	p	0.930		p	0.707		p	0.529
Urea	r	0.052	Folate	r	0.049	RDW-CV	r	−0.134 **
	p	0.230		p	0.403		p	0.002
Creatinine	r	−0.056	Vit. B12	r	−0.001	PLT	r	0.021
	p	0.197		p	0.985		p	0.631
ALT	r	0.075	Chloride	r	−0.063	MPV	r	−0.058
	p	0.087		p	0.225		p	0.188
AST	r	0.032	LDH	r	−0.002	PDW	r	−0.059
	p	0.468		p	0.961		p	0.178
Sodium	r	0.086	T.Protein	r	−0.023	PLT–NötLenf	r	−0.100 *
	p	0.054		p	0.667		p	0.024
Potassium	r	0.078	Albumin	r	0.078	NLR	r	−0.147 **
	p	0.079		p	0.102		p	0.001
Calcium	r	0.165 **	Uric Acid	r	−0.001	PLR	r	−0.068
	p	0.000		p	0.986		p	0.122
Magnesium	r	−0.127 *	Phosphorus	r	0.083	SII	r	−0.082
	p	0.025		p	0.208		p	0.064
Total Bilirubin	r	−0.008	CRP	r	−0.064	CAR	r	−0.063
	p	0.873		p	0.151		p	0.191
Direct Bilirubin	r	0.008	WBC	r	−0.017	AGR	r	0.064
	p	0.867		p	0.701		p	0.245
Indirect Bilirubin	r	0.035	Neutrophil	r	−0.086			
	p	0.474		p	0.053			
ALP	r	0.009	Lymphocyte	r	0.107 *			
	p	0.861		p	0.016			

* *p* < 0.05, ** *p* < 0.01. r: Correlation coefficient.

**Table 5:** Comparison of Clinical. Biopsy. Biochemical. Haematological and Inflammatory Findings of Patients with Positive Carpal Tunnel Syndrome (CTS) According to CTS Aspects.

Variables	CTS Direction Right (*n*:45)	CTS Direction Left (*n*:49)	CTS Direction Bilateral (*n*:171)	*p*
	Mean ± S.S. Med. (Min.-Max.)	Mean ± S.S. Med. (Min.-Max.)	Mean ± S.S. Med. (Min.-Max.)	
Age ^F^	56.04 ± 12.88 59 (6–76)	61.84 ± 9.59 62 (41–82)	60.61 ± 10.43 61 (34–87)	0.089
LDL-K ^F^	144.1 ± 45.53 154 (54–224) a	117.46 ± 38.13 115.5 (46–226) b	114.46 ± 34.44 116 (38–234) b	0.011 *
Chloride ^H^	102.07 ± 2.69 102 (97–107.04) a	104.11 ± 3.36 104 (99–113) b	103.4 ± 3.31 104 (92–113) b	0.030 *
Albumin ^H^	46.19 ± 3.24 46.3 (41.3–53) a	43.43 ± 3.42 43.8 (33.7–52) b	45.02 ± 2.74 45.2 (32–55) a	0.002 **
Uric acid ^H^	5.17 ± 1.25 4.9 (2.76–7.7) a	4.83 ± 1.25 4.92 (2.64–7.27) a	4.64 ± 1.31 4.5 (1.96–9.93) b	0.045 *
Neutrophil ^H^	4.88 ± 2.55 4.23 (1.72–15.52) a	4.01 ± 1.33 3.79 (1.45–8.19) b	3.83 ± 1.41 3.64 (1.75–10.81) b	0.024 *
MCW ^H^	87.37 ± 6.24 88.4 (65.2–99.1) a	85.56 ± 4.97 86 (67.9–99.8) b	87.18 ± 4.97 87.8 (69.8–100.5) a	0.032 *
RDW-CV ^H^	13.98 ± 2.09 1 3.4 (11.9–21.9) a	14.63 ± 1.92 14.15 (12.2–21) b	13.68 ± 1.4 13.5 (11.3–22) a	0.003 **
CAR ^H^	0.13 ± 0.18 0.07 (0.01–0.96)	0.39 ± 1.72 0.05 (0–10.92)	0.12 ± 0.37 0.04 (0–3.94)	0.053

Note: * *p* < 0.05; ** *p* < 0.01. χ^2^: Chi-square test (categorical data); ^F^: One-Way ANOVA test; ^H^: Kruskal–Wallis H test. Lettering indicates statistically significant differences between groups (post-hoc comparisons).

**Table 6:** Evaluation of the Effect of Variables Affecting CTS Positivity with Single Logistic Regression Analysis.

Dependent Variable	Independent Variable	B	S.E.	*p*	Exp(B)/ Odds Rate	Confidence Intervals 95 C.I. for EXP(B)		
						Alt	Üst	R^2^
CTS Positivity	Calcium	0.70	0.18	0.000 **	2.00	1.40	2.88	4.5
CTS Positivity	Magnesium	−1.09	0.59	0.064	0.34	0.11	1.06	2.1
CTS Positivity	Lymphocyte	0.28	0.12	0.022 *	1.32	1.04	1.67	1.4
CTS Positivity	Eosinophil	0.91	0.58	0.115	2.48	0.80	7.67	0.7
CTS Positivity	RBC	0.51	0.16	0.002 **	1.67	1.21	2.29	2.6
CTS Positivity	HGB	0.22	0.05	0.000 **	1.24	1.12	1.38	4.5
CTS Positivity	HCT	0.08	0.02	0.000 **	1.08	1.04	1.12	4.4
CTS Positivity	RDWCV	−0.16	0.05	0.001 **	0.85	0.77	0.94	3.2
CTS Positivity	CompositePlt–NötLenfİndeksi	0.00	0.00	0.022 *	1.00	1.00	1.00	1.6
CTS Positivity	NLR	−0.19	0.06	0.003 **	0.83	0.74	0.94	3.2

* *p* < 0.05, ** *p* < 0.01; EXP(B): Odds Ratio (OR). Probability ratio.

**Table 7:** Results of the ROC Analysis Performed to Predict the Probability of Being a CTS Patient.

Test Variables	AUC	Std. Error	*p*	Cutoff Value	Sensitivity (%)	Specificity (%)	Confidence Interval (95) Lowest Highest	
Composite PLT–Neut Lymph Index	0.56	0.03	0.025 *	425.8	60	50	0.51	0.61
NLR	0.59	0.03	0.001 **	1.69	62	50	0.54	0.64

Note: * *p* < 0.05; ** *p* < 0.01. Area Under the Curve (AUC): The AUC value was used to predict the probability of not having CTS (Carpal Tunnel Syndrome).

## Data Availability

The data reported in the study can be obtained from the corresponding author upon request. Due to confidentiality, the data are not publicly available.

## Abbreviations

The following abbreviations are used in this manuscript:

PPI	Proton pump inhibitors
CTS	Carpal Tunnel Syndrome
ALT	Alanine aminotransferase
AST	Aspartate aminotransferase
TSH	Thyroid-stimulating hormone
EMG	Electromyography
CBC	Complete blood counts
MCV	Mean corpuscular volume
RDW	Distribution width
MPV	Mean platelet volume
PDW	Platelet distribution width
NLR	Neutrophil-to-lymphocyte ratio
PLR	Platelet-to-lymphocyte ratio
SII	Systemic immune inflammation index
CAR	C-reactive protein-to-albumin ratio
AGR	Albumin-to-globulin ratio
ALP	Alkaline phosphatase
GGT	Gamma glutamyl transferase
LDH	Lactate dehydrogenase
